# Supplementary material for: MetaRibo-Seq measures translation in microbiomes
Source: Nat Commun. 2020 Jun 29;11:3268. doi: 10.1038/s41467-020-17081-z (PMC7324362; doi:10.1038/s41467-020-17081-z)
Supplement: Supplementary file 10 — Supplementary Data 7 [file 41467_2020_17081_MOESM10_ESM.zip › File2/Confidence_High_Taxonomy/200745_out.krona.html]

Javascript must be enabled to view this page.

members
magnitude
magnitudeUnassigned
count
unassigned
taxon
rank

200745\_out

24

superkingdom
24
2

phylum
976
24

24
200643
class

171549
24
order

815
1
family

genus
1
816

species

SRS046369\_contig\_number\_1554
1
28116

23
2005525
family

23

SRS016018\_contig\_number\_contig-100\_3544.89572
genus
1
375288

387661
11

SRS015960\_contig\_number\_8645SRS016954\_contig\_number\_15313SRS019601\_contig\_number\_12410SRS019693\_contig\_number\_6951SRS021484\_contig\_number\_14534SRS022609\_contig\_number\_2925SRS043768\_contig\_number\_contig-100\_765.177665SRS048870\_contig\_number\_22888SRS056259\_contig\_number\_contig-100\_5501.137668SRS077127\_contig\_number\_15331SRS104084\_contig\_number\_9772
species


SRS012849\_contig\_number\_25332SRS054352\_contig\_number\_20963SRS055017\_contig\_number\_21188SRS078419\_contig\_number\_34877SRS893366\_contig\_number\_21699
species
5
1750560

species

SRS013476\_contig\_number\_27660SRS017622\_contig\_number\_contig-100\_24.25SRS019968\_contig\_number\_6971SRS076876\_contig\_number\_55SRS1041136\_contig\_number\_14847SRS143070\_contig\_number\_12890
6
46503
